# Association of Coffee Consumption and Its Types According to Addition of Sugar and Creamer with Metabolic Syndrome Incidence in a Korean Population from the Health Examinees (HEXA) Study

**DOI:** 10.3390/nu13030920

**Published:** 2021-03-12

**Authors:** Li-Juan Tan, Hye Joo Jeon, SoHyun Park, Seong-Ah Kim, Kyungjoon Lim, Sangwon Chung, Pahn-Shick Chang, Jong-koo Lee, Daehee Kang, Sangah Shin

**Affiliations:** 1 Department of Food and Nutrition, Chung-Ang University, Gyeonggi-do 17546, Korea; tanlijuan88@cau.ac.kr (L.-J.T.); sohyunp612@gmail.com (S.P.); 2 Department of Agricultural Biotechnology, Seoul National University, Seoul 08826, Korea; hjjeon7@snu.ac.kr (H.J.J.); pschang@snu.ac.kr (P.-S.C.); 3 Department of Urban Society, The Seoul Institute, Seoul 06756, Korea; sakim8864@si.re.kr; 4 Department of Physiology, Anatomy & Microbiology, La Trobe University, Melbourne 3086, Australia; K.Lim@latrobe.edu.au; 5 Korea Food Research Institute, Jeollabuk-do 55365, Korea; schung@kfri.re.kr; 6 Department of Family Medicine, Seoul National University Hospital, Seoul 03080, Korea; docmohw@snu.ac.kr; 7 Department of Preventive Medicine, Seoul National University College of Medicine, Seoul 03080, Korea; dhkang@snu.ac.kr

**Keywords:** coffee consumption, metabolic syndrome, Korean adults, health examinee study, cohort study

## Abstract

Coffee is widely consumed worldwide, and numerous studies indicate that coffee consumption may potentially affect the development of chronic diseases. Metabolic syndrome (MetS) may constitute a risk factor for chronic diseases. We aimed to prospectively evaluate the association between coffee consumption and MetS incidence. All participants were selected from the Health Examinees study. MetS was defined by the Adult Treatment Panel III criteria of the National Cholesterol Education Program. A multivariate Cox proportional hazards regression model was used to assess the relationship between coffee consumption and MetS incidence. In comparison with non-consumers, male moderate consumers (≤3 cups/day) showed a lower risk for low high-density lipoprotein cholesterol (HDL-C) (≤1 cup/day, hazard ratio (HR): 0.445, 95% confidence interval (CI): 0.254–0.780; 1–3 cups/day, HR: 0.507, 95% CI: 0.299–0.859) and high fasting blood glucose (FPG) (≤1 cup/day, HR: 0.694, 95% CI: 0.538–0.895; 1–3 cups/day, HR: 0.763, 95% CI: 0.598–0.972). Male 3-in-1 coffee (coffee with sugar and creamer) consumers also showed a lower risk for low HDL-C (HR: 0.423, 95% CI: 0.218–0.824) and high FPG (HR: 0.659, 95% CI: 0.497–0.874). These findings indicate a negative association between moderate coffee consumption and low HDL-C and high FPG among Korean male adults.

## 1. Introduction

In the modern era, coffee is widely consumed globally in everyday life. The International Coffee Organization announced in its 2016 report that the global coffee consumption had attained an average annual growth rate of 2% since 2012, maintaining 152.1 million bags of coffee in 2015. The Asian market has shown the strongest growth in recent years at an average consumption rate of 5.2%, which is above the global average [1]. In South Korea, coffee is one of the most frequently consumed beverages, and the consumption rate is increasing [2]. Previous studies have reported that coffee contains numerous bioactive compounds including caffeine and polyphenols [3,4,5,6]. Polyphenols are well-known antioxidants and have preventive effects on various chronic diseases, such as cardiovascular diseases, cancers, and diabetes [7,8,9].

Metabolic syndrome (MetS) is a group of metabolic disorders that are closely linked to the development of cardiovascular disease, diabetes, and strokes [10]. The prevalence of MetS is increasing rapidly in Korea [11,12]. Importantly, the prevalence of MetS among the Korean adults was 20.3%, and approximately 40% of adults aged over 60 years had MetS [13]. In this regard, lifestyle and dietary components are strongly associated with MetS prevention [14,15,16,17,18]. In particular, MetS occurrence is reportedly associated with coffee consumption [18].

A cross-sectional study based on the Japanese general population suggested that coffee consumption was associated with a lower number of MetS components, possibly due to the role of caffeine derived from coffee in reducing insulin resistance [19]. Another Japanese study also showed that in male participants, moderate coffee consumption (≥4 cups/day) had an inverse association with MetS, especially in regard to high blood pressure and high triglyceride levels [20]. In Korea, a cross-sectional study on stroke suggested that high-frequency coffee consumption was associated with a lower prevalence of hypertension, diabetes mellitus, and hyperlipidemia [2]. Lee et al. suggested that adequate caffeine intake (approximately 45 mg/day) was associated with a lower prevalence of diabetes and hypertriglyceridemia in healthy Korean adults [21]. We also showed the aforementioned association in our previous cross-sectional study. In the Health Examinees (HEXA) study, we revealed that coffee consumption, regardless of coffee types, reduced MetS prevalence among Korean adults [22]. However, the longitudinal association between coffee consumption and MetS occurrence could not be proved in our previous study, and the effects of long-term coffee consumption on the development of MetS and its components remain unclear. Hence, in the current study, we aimed to prospectively evaluate the association between coffee consumption and MetS prevention. At the same time, we also aimed to explore the impact of changes in coffee consumption patterns on MetS during the follow-up periods.

## 2. Materials and Methods

### 2.1. Study Population

The HEXA study is a large-scale genomic cohort study of Korean adults (age ≥40 years). At baseline, the HEXA study included 173,357 participants who underwent a health check-up and completed a series of questionnaires from 2004 to 2013, and a follow-up HEXA study included 65,642 participants who were engaged between 2012 and 2016. A previous study revealed more details about the HEXA study [23].

**Figure 1:**
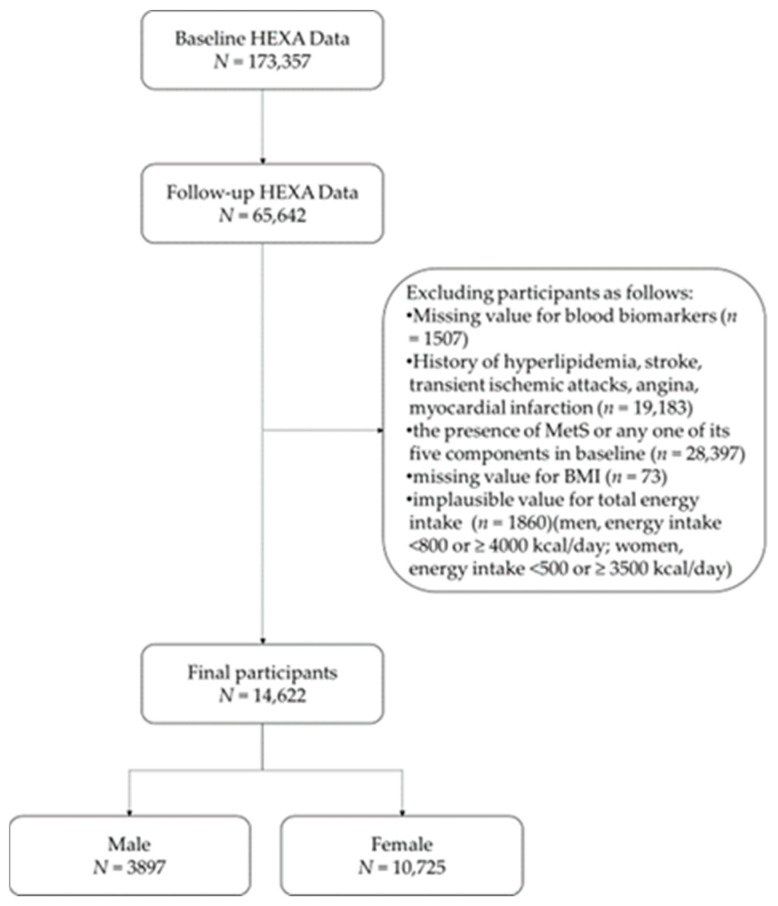
Selection flowchart of study participants. BMI, body mass index; HEXA, Health Examinees; MetS, metabolic syndrome.

In the current study, we excluded 51,020 (from 65,642) participants who met the exclusion criteria (missing biomarker values, a history of related disease, the presence of MetS or any of its five components at baseline, missing body mass index (BMI) value, and implausible total energy intake values (men, total energy intake <800 or ≥4000 kcal/day; women, total energy intake <500 or ≥3500 kcal/day [22])). Consequently, 14,622 participants (male, *N* = 3897; female, *N* = 10,725) were included in this study (Figure 1).

### 2.2. Assessment of Coffee Consumption

The amount of coffee consumption was assessed as part of a self-administered food-frequency questionnaire (FFQ) that was designed to capture the usual dietary intake of Koreans [2,21]. The reproducibility and validity of the FFQ were previously evaluated [24,25]. Participants were asked how often, on average, they consumed coffee over the previous 12 months before baseline and follow-up sample collection. Participants could choose intake frequency and the average amount of coffee intake per drink from three categories (“1/2 cup”, “1 cup”, and “2 cups”). Participants were also asked the portion sizes of sugar and creamer if these were added into the coffee. Based on the average of two observations, the total amount of coffee consumption was categorized into 0, ≤1, 1–3 (>1 to ≤3 cups/day), and >3 cups/day [2].

The participants’ style of coffee consumption was also analyzed (non-consumer, black coffee consumer, 3-in-1 coffee consumer, and others) according to coffee additives. People who did not drink coffee, both at baseline and follow-up, were classified as non-consumers. Coffee consumers, both at baseline and follow-up, who added or did not add sugar and creamer, were classified as 3-in-1 and black coffee consumers, respectively. All remaining coffee consumers were classified as “others”.

In order to further evaluate the relationship between coffee consumption and MetS incidence, we also categorized coffee consumption habits into four groups: “non–non” “non–coffee”, “coffee–non”, and “coffee–coffee”. Participants who were non-consumers both at baseline and at follow-up were classified as “non–non”. Participants who drank coffee only at follow-up were classified as “non–coffee”. Participants who drank coffee only at baseline were classified as “coffee–non”. Participants who drank coffee both at baseline and at follow-up were classified as “coffee–coffee”.

### 2.3. Definition of Metabolic Syndrome

According to the modified Asian version of the Adult Treatment Panel III criteria of the National Cholesterol Education Program (NCEP-ATP III), MetS was defined if three or more of the following five traits were present: (1) abdominal obesity, waist circumference ≥90 cm in men and ≥80 cm in women; (2) high triglyceride (TG), serum TG ≥150 mg/dL; (3) low high-density lipoprotein (HDL)-cholesterolemia, serum HDL-C <40 mg/dL in men and <50 mg/dL in women; (4) high blood pressure, systolic blood pressure ≥130 mmHg or diastolic blood pressure ≥85 mmHg; and (5) high fasting plasma glucose (FPG), FPG ≥100 mg/dL [11,12].

### 2.4. Other Variables

Using the questionnaire, participants were asked about sociodemographic parameters, medical history, medication use, family history, physical activity, alcohol intake, and smoking habits. Physical activity levels were evaluated as “active” if participants engaged in exercises for over 30 min twice a week [26]. Regarding smoking status, participants were categorized into three groups based on the responses to a question such as “Have you smoked more than 20 packs (400 cigarettes) so far?”. If the answer was “never”, the participants were classified as “non-smokers”. If the participants answered “yes” and were still smoking at the time of the survey, they were classified as “current smokers”. If the participants answered “yes” but had quit smoking at the time of the survey, they were classified as “past smokers”. All participants responded to “Are you unable to drink or refuse to do for religious or other reasons” in the survey. In the current analysis, if the answer was “yes”, we classified the participant as “non-drinker”; the rest of the participants were classified as “drinker”. Age was categorized into “40–49”, “50–59”, “60–69”, and “70–79” years. BMI was calculated as weight (kg) divided by the square of height (m^2^) [27]. We divided participants into three BMI groups: <18.5 kg/m^2^, 18.5–25 kg/m^2^, >25 kg/m^2^. Educational level was classified into three levels: under middle school, high school, and college or above.

### 2.5. Statistical Analysis

All analyses in this prospective study were stratified by sex. In the descriptive analysis of general characteristics according to coffee consumption, categorical variables (educational level, current drinking status, current smoking status, and physical activity) were presented as numbers and percentages (n, %) and the chi square test was used for calculating *p* values. Continuous variables (age, BMI, and energy intake) were presented as mean ± standard error; their *p* values were analyzed by the generalized linear model and the significant analysis of variance of each coffee consumption group was carried out with a Duncan test. In this study, all coffee variables and confounding variables were fixed effects. A multivariate Cox proportional hazards regression model was used to estimate all hazard ratios (HRs) and 95 % confidence intervals (CIs) for men and women separately between coffee intake and MetS occurrence. The model was adjusted for all aforementioned continuous variables and categorical variables. Tests for trends were performed using a generalized linear model with the median value of each group; median values were modeled as a continuous variable in the model. Statistical significance was defined as a *p* value <0.05. All analyses were performed using SAS program version 9.4 (SAS Institute Inc., Cary, NC, USA).

## 3. Results

The total percentages of coffee drinkers in our study were 93% in men and 89% in women. Participant characteristics in the HEXA study based on coffee type are shown in Table 1. Participant characteristics, based on coffee consumption and coffee intake pattern change, are shown in Supplementary Tables S1 and S2, respectively. Male “3-in-1” and “other” coffee consumers were approximately 91% of male participants; for female participants, this proportion corresponded to approximately 84%. Most participants consumed less than 3 cups of coffee/day (75% in men, and 80% in women). Participants consuming more coffee tended to be younger, smokers, alcohol consumers, more physically active, and with higher BMI values. Moreover, participants who consumed more 3-in-1 coffee tended to be younger, smokers, alcohol consumers, and with higher BMI values.

The incidences of MetS were 5.1% and 3.2% in male and female participants, respectively. The adjusted HRs (95% CIs) of MetS and its five components according to the types of coffee consumed, amount of coffee consumed, and change of coffee intake pattern are presented in Table 2 and Table 3, respectively. Although there was no significant difference between MetS occurrence and coffee consumption, some MetS components showed a statistical link with coffee consumption. After adjusting for potential confounders, male participants with moderate coffee consumption (≤3 cups/day) showed a lower risk for low HDL-C (≤1 cup/day, HR: 0.445, 95% CI: 0.254–0.780; 1–3 cups/day, HR: 0.507, 95% CI: 0.299–0.859) and high FPG (≤1 cups/day, HR: 0.694, 95% CI: 0.538–0.895; 1–3 cups/day, HR: 0.763, 95% CI: 0.598–0.972). Female participants with higher coffee consumption showed an increasing trend in risks for abdominal obesity (*p* for trend = 0.0043), high blood pressure (*p* for trend <0.0001), and high FPG (*p* for trend <0.0001). Female heavy coffee consumer (>3 cups/day) had a higher risk for high blood pressure (HR:1.588, 95% CI: 1.260–2.000) and high FPG (HR:1.526, 95% CI: 1.193–1.953). Compared to non–coffee drinkers, male participants consuming 3-in-1 coffee and other coffee showed a lower risk for low HDL-C (3-in-1 coffee, HR: 0.423, 95% CI: 0.218–0.824; other coffee, HR: 0.493, 95% CI: 0.298–0.817) and high FPG (3-in-1 coffee, HR: 0.659, 95% CI: 0.497–0.874; other coffee, HR: 0.749, 95% CI: 0.592–0.949). Female participants consuming black coffee showed a higher risk for high blood pressure (HR: 1.442, 95% CI: 1.094–1.900) and high FPG (HR: 1.558, 95% CI: 1.167–2.081). Since the “other” group is much larger than the other three groups, we further subdivided the “other” group into ≤1 teaspoon group both at baseline and at follow-up, but we have produced consistent results (Supplementary Materials, Table S3). Among male participants, 3-in-1 coffee consumers still have a lower risk for low HDL-C and high FPG compared to non-consumers. Among female participants, black coffee consumers still have a higher risk for high FPG. In addition to the relationship between “additives ≤1 teaspoon” and blood pressure among women participants, the two subgroups of the “other” group showed the same relationship with MetS and its components as the “other” group.

The association between change of coffee consumption pattern and MetS incidence is shown in Table 4. We found that, among male participants, those in the non–coffee and coffee–coffee groups showed a lower risk for low HDL-cholesterol (non–coffee, HR: 0.379, 95% CI: 0.169–0.849; coffee–coffee, HR: 0.488, 95% CI: 0.293–0.811) and high fasting plasma glucose (non–coffee, HR: 0.683, 95% CI: 0.495–0.941; coffee–coffee, HR: 0.754, 95% CI: 0.595–0.955) than those in the non–non group. However, we found no significant differences in these parameters among female participants.

## 4. Discussion

Previous studies have shown the cross-sectional association between coffee consumption and the risk of developing MetS in the Korean population [22,28,29,30]. However, there is a paucity of data pertaining to the longitudinal association between coffee consumption and MetS occurrence among Korean adults.

In the current study, the incidences of MetS in men and women were 5.1% and 3.2%, respectively. Coffee consumption had no association with the development of MetS in the general Korean adult population (age ≥40 years) as compared to coffee non-consumers, regardless of the amount or type (“black”, “3-in-1”, and “others”) of consumption. However, there were significant associations between coffee and some components of MetS. Among male participants, serum HDL-C and FPG levels were associated with coffee consumption. Somewhat differently, blood pressure and serum FPG levels were associated with coffee consumption among female participants.

Coffee-derived phenolic acids, due to their antioxidant properties, could enhance HDL-mediated cholesterol efflux from macrophages [31]. Furthermore, in men, short-term coffee consumption can increase the serum HDL-C levels [32]. In the present study, we found that male participants with moderate coffee consumption (≤3 cups/day) had a significantly lower risk of developing low HDL-cholesterol compared to non–coffee consumers (Table 2). Our findings corroborated with those of Kempf et al. whose single-blind 3-test clinical trial showed the impact of coffee in raising HDL-C levels [33]. Moreover, after analyzing the association between coffee pattern change and MetS incidence, we also found that current coffee-consumers (“non–coffee” and “coffee–coffee”) had a lower risk of developing low HDL-cholesterol among male participants compared to non-consumers, regardless of coffee type (Table 3 and Table 4).

Caffeine, another main bioactive compound in coffee, has numerous biological impacts on human health [34]. The plasma glucose regulating effect of caffeine remains unclear. In our study, male moderate consumers (<3 cups/day) showed a lower risk of developing high FPG. However, female participants showed an opposite relationship, with respect to that of male participants, between coffee consumption and FPG. A previous study showed that short-term coffee consumption could decrease insulin sensitivity and impair glucose tolerance due to the caffeine-blocking effect of the A1 receptor (a pattern recognition receptor with anti-inflammatory effect in cardiovascular disease); however, the effect is temperate [35,36]. Long-term coffee consumption protects against the development of type 2 diabetes [37] and affects post-load rather than fasting glucose metabolism [38]. Furthermore, the impact of caffeinated or decaffeinated coffee consumption on plasma glucose depended on the glycemic index of a meal [39].

Robertson et al. indicated that different genotypes (mainly rs762551 single-nucleotide polymorphism in the *CYP1A2* gene can affect the rate of caffeine metabolism) might influence the plasma glucose level [40]. In this regard, environmental and constitutional factors have a great influence on *CYP1A2* activity, including smoking status, sex, and race [41]. Gunes et al. suggested that *CYP1A2* activity was lower in women. This may be an explanation for the abovementioned opposite association showed by female and male participants. Alternatively, lifestyle differences between men and women may be the key to our findings. The degree of physical activity, sleep duration, menopause condition, alcohol consumption, and diet patterns could influence MetS incidence [14,42,43,44]. Moreover, we have observed behavioral differences in drinking and smoking between male and female participants (Table 1) in this study.

In Korea, coffee drinkers prefer to drink coffee with sugar or creamer, especially the middle aged or elderly population [45,46]. Instant coffee with sugar or creamer accounts for 80–90% of coffee consumed in the Korean coffee market [47]. Concerns have been raised over whether the increase in saturated fat or simple sugar intake from creamer or sugar in 3-in-1 coffee may be harmful to health; hitherto, no clear findings have been reported, which makes creamer or sugar controversial [48]. Our results suggested that, among men, 3-in-1 or other-coffee consumers had a lower risk of developing high FPG and low HDL-cholesterol than non-consumers (Table 3). A study on the South Korean coffee market showed that the main fatty acid from coffee creamer is lauric acid [49]. Lauric acid is a kind of medium-chain fatty acid, which is mainly used as a substrate for the synthesis of apolipoprotein (apo)A1 and apoB to promote the particle formation of HDL-C [50].

Estrogen has antioxidant properties that play a cardioprotective role [51]. In postmenopausal women, estrogen secretion decreases, leading to increase oxidative stress. Oxidative stress is an important mechanism of cardiovascular disease, especially atherosclerosis, and is associated with differences in sex [51]. Yeasmin et al. suggested that lower estrogen levels could cause hypertension in postmenopausal women [52]; moreover, regular coffee consumption may be harmful to hypertension-prone individuals [53]. Approximately half (51.68%) of the female participants in our study were postmenopausal. The reduced estrogen secretion of these participants may have affected our findings that female participants, who drank black coffee were at risk of high blood pressure development.

The self-reported coffee consumption, inevitably, biased exposure factor assessment. However, such bias would have little or no impact on the results of large-scale prospective studies. The 106-item semi-FFQ has been validated by previous studies [24,25]. In the current study, we simply classified drinking status and physical activity; we did not categorize the amount of alcohol consumption and the level of physical activity in detail, which could impact MetS. However, in our study, drinking status and physical activity were not the exposure factors, and as confounding variables they have been adjusted in the model. In the analysis of the change of coffee consumption pattern, we defined the change of pattern according to the responses at the time of baseline and follow-up survey. However, as the average follow-up period was about five years, we were unable to fully document the pattern change of coffee consumption outside the period of survey. Another limitation is that we did not adjust for the consumption of cola and tea, which contain caffeine and polyphenols. In Korea, coffee has surpassed rice and kimchi as the most consumed food [30], and in our analysis, the consumptions of cola and tea were very small, and their impacts can be ignored. In HEXA data, there is no information about coffee brewing methods, types of coffee bean, and caffeine content in one typical coffee. Therefore, although we have suggested an association between coffee consumption and MetS, we cannot identify which compounds derived from coffee have an impact on MetS.

## 5. Conclusions

Our study showed a statistically significant negative association between regular coffee intake and the incidence of low HDL cholesterol and high FPG among Korean male adults, regardless of the coffee type. However, we did not find the same relationship among women. We suggest that there is a sex-specific effect of coffee consumption on the incidence of low HDL-cholesterol and high FPG among adults in Korea. Importantly, this leads us to our future studies wherein metabolic components will be analyzed to investigate the effect of coffee-derived compounds on men and women.

## Figures and Tables

**Table 1:** Participant general characteristics based on consumed coffee type and sex ^1^.

	Coffee Type				
	None	Black	3-in-1	Others	*p* Value
Men (*N* = 3897)					
*N* (incidence %)	276 (3.99%)	90 (3.33%)	610 (5.41%)	2921 (5.20%)	
Age (years)	55.69 ± 0.50 ^a^	53.94 ± 0.94 ^b^	52.37 ± 0.33 ^c^	53.71 ± 0.16 ^bc^	0.0006
40–49	68 (5.16%)	31 (2.35%)	243 (18.42%)	977 (74.07%)	0.0004
50–59	107 (7.28%)	31 (2.11%)	233 (15.86%)	1098 (74.74%)	
60–69	95 (9.07%)	26 (2.48%)	127 (12.13%)	799 (76.31%)	
70–79	6 (9.68%)	2 (3.23%)	7 (11.29%)	47 (75.81%)	
BMI (kg/m^2^)	21.85 ± 0.13 ^b^	22.89 ± 0.22 ^a^	22.91 ± 0.09 ^a^	22.68 ± 0.04 ^a^	<0.0001
<18.5	23 (8.33%)	2 (2.22%)	15 (2.46%)	95 (3.25%)	<0.0001
18.5–25	227 (82.25%)	80 (88.89%)	472 (77.38%)	2328 (79.70%)	
≥25	26 (9.42%)	8 (8.89%)	123 (20.16%)	498 (17.05%)	
Waist circumference (cm)	79.00 ± 0.36 ^b^	80.37 ± 0.54 ^a^	80.84 ± 0.21 ^a^	80.37 ± 0.10 ^a^	0.0003
Smoking					<0.0001
Non-smoker	145 (52.54%)	34 (37.78%)	223 (36.56%)	982 (33.62%)	
Past smoker	110 (39.86%)	32 (35.56%)	223 (36.56%)	1132 (38.75%)	
Current smoker	18 (6.52%)	24 (26.67%)	162 (26.56%)	795 (27.22%)	
Physical activity (yes, %)	128 (46.38%)	38 (42.22%)	244 (40.00%)	1142 (39.10%)	0.1440
Educational level					<0.0001
Under middle school	49 (17.75%)	17 (18.89%)	65 (10.66%)	627 (21.47%)	
High school	106 (38.41%)	33 (36.67%)	208 (34.10%)	1118 (38.27%)	
College or above	117 (42.39%)	40 (44.44%)	333 (54.59%)	1149 (39.34%)	
Alcohol consumption (yes, %)	150 (54.35%)	64 (71.11%)	472 (77.38%)	2112 (72.30%)	<0.0001
Biomarkers					
TG (mg/dL)	83.42 ± 1.72 ^b^	91.16 ± 3.34 ^a^	88.48 ± 1.15 ^ab^	87.94 ± 0.56 ^ab^	0.1802
HDL-C (mg/dL)	54.80 ± 0.69 ^a^	55.12 ± 1.15 ^a^	55.21 ± 0.41 ^a^	54.60 ± 0.20 ^a^	0.6240
FPG (mg/dL)	87.92 ± 0.42 ^a^	88.06 ± 0.82 ^a^	87.95 ± 0.28 ^a^	88.28 ± 0.12 ^a^	0.5657
SBP (mmHg)	115.05 ± 0.51 ^a^	114.59 ± 0.86 ^a^	114.91 ± 0.34 ^a^	114.74 ± 0.16 ^a^	0.8810
DBP (mmHg)	72.53 ± 0.42 ^a^	72.37 ± 0.66 ^a^	72.79 ± 0.27 ^a^	72.18 ± 0.13 ^a^	0.3093
Women (*N* = 10,725)					
N (incidence %)	1119 (3.13%)	548 (2.55%)	1130 (2.39%)	7928 (3.33%)	
Age (years)	52.37 ± 0.21 ^a^	48.63 ± 0.29 ^bc^	48.44 ± 0.19 ^c^	49.08 ± 0.07 ^b^	<0.0001
40–49	401 (6.95%)	319 (5.53%)	669 (11.60%)	4379 (75.92%)	<0.0001
50–59	523 (12.93%)	184 (4.55%)	390 (9.64%)	2948 (72.88%)	
60–69	188 (21.20%)	44 (4.96%)	68 (7.67%)	587 (66.18%)	
70–79	7 (28.00%)	1 (4.00%)	3 (12.00%)	14 (56.00%)	
BMI (kg/m^2^)	21.20 ± 0.06 ^c^	21.62 ± 0.08 ^b^	21.90 ± 0.06 ^a^	21.74 ± 0.02 ^ab^	<0.0001
<18.5	97 (8.67%)	24 (4.38%)	35 (3.10%)	382 (4.82%)	<0.0001
18.5–25	980 (87.58%)	487 (88.87%)	985 (87.17%)	6902 (87.06%)	
≥25	42 (3.75%)	37 (6.75%)	110 (9.73%)	644 (8.12%)	
Waist circumference (cm)	71.59 ± 0.15 ^b^	80.37 ± 0.54 ^b^	72.13 ± 0.14 ^a^	71.83 ± 0.05 ^ab^	0.0046
Smoking status					0.0005
Non-smoker	1107 (98.93%)	523 (95.44%)	1104 (97.70%)	7670 (96.75%)	
Past smoker	5 (0.45%)	10 (1.82%)	5 (0.44%)	78 (0.98%)	
Current smoker	5 (0.45%)	13 (2.37%)	20 (1.77%)	140 (1.77%)	
Physical activity (yes, %)	476 (42.54%)	233 (42.52%)	413 (36.55%)	3155 (39.80%)	0.0092
Educational level					<0.0001
Under middle school	339 (30.29%)	106 (19.34%)	155 (13.72%)	1619 (20.42%)	
High school	492 (43.97%)	267 (48.72%)	561 (49.65%)	3933 (49.61%)	
College or above	272 (24.31%)	174 (31.75%)	405 (35.84%)	2316 (29.21%)	
Alcohol consumption (yes, %)	174 (15.55%)	232 (42.34%)	466 (41.24%)	3032 (38.24%)	<0.0001
Biomarkers					
TG (mg/dL)	77.43 ± 0.84 ^a^	74.07 ± 1.09 ^b^	73.31 ± 0.84 ^b^	74.91 ± 0.31 ^b^	0.0636
HDL-C (mg/dL)	63.41 ± 0.31 ^b^	64.83 ± 0.46 ^a^	64.06 ± 0.31 ^ab^	64.20 ± 0.12 ^ab^	0.3941
FPG (mg/dL)	86.13 ± 0.21 ^a^	86.00 ± 0.29 ^a^	85.86 ± 0.20 ^a^	86.02 ± 0.08 ^a^	0.8706
SBP (mmHg)	111.63 ± 0.30 ^a^	110.99 ± 0.40 ^ab^	110.64 ± 0.28 ^b^	111.15 ± 0.11 ^ab^	0.3168
DBP (mmHg)	69.66 ± 0.22 ^a^	68.79 ± 0.30 ^b^	69.45 ± 0.22 ^a^	69.47 ± 0.08 ^a^	0.1263

^1^*p* values of continuous variables (presented as mean ± standard error) were calculated using general linear models, and *p* values of categorical variables (presented as n (%)) were calculated using the chi-square test. Statistical significance was defined as a *p* value <0.05. The different alphabets in same row show significant difference of 0.05 based on Duncan test. Physical inactivity was defined as performing over 30 min of exercise less than twice a week. BMI, body mass index; TG, triglyceride; HDL-C, high-density lipoprotein cholesterol; FPG, fasting plasma glucose; SBP, systolic blood pressure; DBP, diastolic blood pressure.

**Table 2:** The association between total coffee consumption and the incidence of MetS and its five components (reference group: non–coffee consumers) ^1^.

	Coffee Consumption, Cups/Day				*p* for Trend
	0	≤1	1–3	>3	
Men (*N* = 3897)	276	1071	1853	697	
Median, range (cups/day)	0, 0–0	0.75, 0.01–1.00	1.50, 1.04–3.00	3.50, 3.25–8.50	
MetS	11 (3.99%)	53 (4.95%)	101 (5.45%)	34 (4.88%)	
	Ref	0.771 (0.399–1.491)	0.823 (0.437–1.550)	0.620 (0.305–1.258)	0.1844
Abdominal obesity	16 (5.80%)	78 (7.28%)	188 (10.15%)	68 (9.76%)	
	Ref	0.870 (0.503–1.504)	1.207 (0.717–2.031)	1.120 (0.634–1.978)	0.3118
High triglyceride	24 (8.70%)	123 (11.48%)	263 (14.19%)	101 (14.49%)	
	Ref	0.975 (0.628–1.514)	1.117 (0.732–1.704)	0.987 (0.625–1.559)	0.8609
High blood pressure	66 (23.91%)	258 (24.09%)	475 (25.63%)	165 (23.67%)	
	Ref	0.788 (0.600–1.035)	0.874 (0.673–1.136)	0.855 (0.636–1.149)	0.9718
Low HDL-cholesterol	18 (6.52%)	41 (3.83%)	74 (3.99%)	36 (5.16%)	
	Ref	0.445 (0.254–0.780)	0.507 (0.299–0.859)	0.611 (0.335–1.113)	<0.0001
High fasting plasma glucose	78 (28.26%)	271 (25.30%)	505 (27.25%)	192 (27.55%)	
		0.694 (0.538–0.895)	0.763 (0.598–0.972)	0.783 (0.596–1.030)	0.9599
Women (*N* = 10,725)	1119	3390	5234	982	
Median, range (cups/day)	0, 0–0	0.75, 0.01–1.00	1.50, 1.07–3.00	3.50, 3.25–8.50	
MetS	35 (3.13%)	99 (2.92%)	171 (3.27%)	35 (3.56%)	
	Ref	0.703 (0.475–1.041)	0.935 (0.639–1.367)	1.200 (0.730–1.972)	0.0509
Abdominal obesity	164 (14.66%)	624 (18.41%)	985 (18.82%)	192 (19.55%)	
	Ref	0.974 (0.818–1.159)	1.108 (0.934–1.315)	1.234 (0.992–1.535)	0.0043
High triglyceride	96 (8.58%)	277 (8.17%)	377 (7.20%)	58 (5.91%)	
	Ref	0.838 (0.662–1.061)	0.850 (0.673–1.074)	0.720 (0.511–1.013)	0.1173
High blood pressure	160 (14.30%)	463 (13.66%)	739 (14.12%)	160 (16.29%)	
	Ref	0.885 (0.737–1.062)	1.151 (0.964–1.374)	1.588 (1.260–2.000)	<0.0001
Low HDL-cholesterol	83 (7.42%)	233 (6.87%)	348 (6.65%)	55 (5.60%)	
	Ref	0.828 (0.642–1.068)	0.977 (0.762–1.252)	0.883 (0.618–1.261)	0.8671
High fasting plasma glucose	135 (12.06%)	451 (13.30%)	697 (13.32%)	145 (14.77%)	
	Ref	1.027 (0.845–1.248)	1.248 (1.032–1.510)	1.526 (1.193–1.953)	<0.0001

^1^ Incidence was presented as n (%), and hazard ratios and 95 % confidence intervals were calculated using Cox model after adjusting for continuous (age, BMI, and energy intake) and categorical (educational level, current drinking status, current smoking status, and physical activity) variables. *p* for trends were performed using a generalized linear model. MetS, metabolic syndrome; BMI, body mass index; HDL, high-density lipoprotein. High triglyceride, serum triglycerides ≥150 mg/dL; Low HDL-cholesterol, serum HDL-cholesterol <40 mg/dL in men and <50 mg/dL in women; Abdominal obesity, waist circumference ≥90 cm in men and ≥80 cm in women; High fasting plasma glucose, fasting plasma glucose ≥100 mg/dL; High blood pressure, systolic blood pressure ≥130 mmHg or diastolic blood pressure ≥85 mmHg.

**Table 3:** Association between coffee type and MetS and its five components (reference group: non–coffee consumers) ^1^.

	COFFEE TYPE			
	None	Black	3-in-1	Others
Men (*N* = 3897)	276	90	610	2921
MetS	11 (3.99%)	3 (3.33%)	33 (5.41%)	152 (5.20%)
	Ref	0.649 (0.179–2.357)	0.806 (0.398–1.635)	0.776 (0.416–1.448)
Abdominal obesity	16 (5.80%)	6 (6.67%)	52 (8.52%)	276 (9.45%)
	Ref	1.001 (0.388–2.583)	1.011 (0.566–1.807)	1.093 (0.654–1.826)
High triglyceride	24 (8.70%)	14 (15.56%)	82 (13.44%)	391 (13.39%)
	Ref	1.609 (0.829–3.121)	1.040 (0.654–1.654)	1.038 (0.685–1.574)
High blood pressure	66 (23.91%)	30 (33.33%)	148 (24.26%)	720 (24.65%)
	Ref	1.519 (0.984–2.343)	0.848 (0.630–1.142)	0.823 (0.637–1.063)
Low HDL-cholesterol	18 (6.52%)	7 (7.78%)	19 (3.11%)	125 (4.28%)
	Ref	1.358 (0.561–3.288)	0.423 (0.218–0.824)	0.493 (0.298–0.817)
High fasting plasma glucose	78 (28.26%)	24 (26.67%)	147 (24.10%)	797 (27.29%)
	Ref	1.008 (0.637–1.596)	0.659 (0.497–0.874)	0.749 (0.592–0.949)
Women (*N* = 10,725)	1119	548	1130	7928
MetS	35 (3.13%)	14 (2.55%)	27 (2.39%)	264 (3.33%)
	Ref	0.652 (0.335–1.269)	0.653 (0.390–1.093)	0.864 (0.600–1.243)
Abdominal obesity	164 (14.66%)	91 (16.61%)	212 (18.76%)	1498 (18.90%)
	Ref	0.946 (0.721–1.240)	1.077 (0.874–1.327)	1.055 (0.895–1.244)
High triglyceride	96 (8.58%)	35 (6.39%)	79 (6.99%)	598 (7.54%)
	Ref	0.952 (0.642–1.411)	0.798 (0.588–1.084)	0.836 (0.670–1.045)
High blood pressure	160 (14.30%)	77 (14.05%)	165 (14.60%)	1120 (14.13%)
	Ref	1.442 (1.094–1.900)	1.111 (0.888–1.388)	1.019 (0.860–1.208)
Low HDL-cholesterol	83 (7.42%)	32 (5.84%)	72 (6.37%)	532 (6.71%)
	Ref	1.085 (0.717–1.641)	0.895 (0.647–1.237)	0.894 (0.705–1.135)
High fasting plasma glucose	135 (12.06%)	73 (13.32%)	142 (12.57%)	1078 (13.60%)
	Ref	1.558 (1.167–2.081)	1.102 (0.866–1.403)	1.147 (0.955–1.378)

^1^ Incidence was presented as n (%), and hazard ratios and 95% confidence intervals were calculated using Cox model after adjusting for continuous (age, BMI, and energy intake) and categorical (educational level, current drinking status, current smoking status, and physical activity) variables. MetS, metabolic syndrome; BMI, body mass index; HDL, high-density lipoprotein. High triglyceride, serum triglycerides ≥150 mg/dL; Low HDL-cholesterol, serum HDL-cholesterol <40 mg/dL in men and <50 mg/dL in women; Abdominal obesity, waist circumference ≥90 cm in men and ≥80 cm in women; High fasting plasma glucose, fasting plasma glucose ≥100 mg/dL; High blood pressure, systolic blood pressure ≥130 mmHg or diastolic blood pressure ≥85 mmHg.

**Table 4:** The association between the change of coffee consumption pattern and incidence of MetS and its five components (reference group: non–non consumers) ^1^.

	Non–Non	Non–Coffee	Coffee–Non	Coffee–Coffee
Men (*N* = 3897)	276	262	200	3159
MetS	11 (3.99%)	18 (6.87%)	11 (5.50%)	159 (5.03%)
	Ref	1.241 (0.582–2.649)	0.941 (0.397–2.229)	0.724 (0.388–1.354)
Abdominal obesity	16 (5.80%)	22 (8.40%)	13 (6.50%)	299 (9.47%)
	Ref	1.208 (0.629–2.321)	0.895 (0.421–1.904)	1.085 (0.648–1.815)
High triglyceride	24 (8.70%)	35 (13.36%)	23 (11.50%)	429 (13.58%)
	Ref	1.063 (0.631–1.791)	1.063 (0.599–1.888)	1.048 (0.691–1.589)
High blood pressure	66 (23.91%)	67 (25.57%)	50 (25.00%)	781 (24.72%)
	Ref	0.745 (0.529–1.049)	0.839 (0.578–1.217)	0.853 (0.660–1.101)
Low HDL-cholesterol	18 (6.52%)	9 (3.44%)	13 (6.50%)	129 (4.08%)
	Ref	0.379 (0.169–0.849)	0.832 (0.405–1.709)	0.488 (0.293–0.811)
High fasting plasma glucose	78 (28.26%)	73 (27.86%)	47 (23.50%)	848 (26.84%)
	Ref	0.683 (0.495–0.941)	0.665 (0.461–0.959)	0.754 (0.595–0.955)
Women (*N* = 10,725)	1119	697	729	8180
MetS	35 (3.13%)	19 (2.73%)	23 (3.16%)	263 (3.22%)
	Ref	0.715 (0.408–1.251)	0.821 (0.483–1.396)	0.856 (0.593–1.237)
Abdominal obesity	164 (14.66%)	127 (18.22%)	129 (17.70%)	1545 (18.89%)
	Ref	1.027 (0.814–1.295)	1.000 (0.792–1.261)	1.064 (0.902–1.257)
High triglyceride	96 (8.58%)	61 (8.75%)	55 (7.54%)	596 (7.29%)
	Ref	0.943 (0.683–1.301)	0.761 (0.545–1.062)	0.834 (0.666–1.043)
High blood pressure	160 (14.30%)	103 (14.78%)	108 (14.81%)	1151 (14.07%)
	Ref	0.978 (0.763–1.254)	0.935 (0.732–1.195)	1.070 (0.901–1.270)
Low HDL-cholesterol	83 (7.42%)	44 (6.31%)	54 (7.41%)	538 (6.58%)
	Ref	0.812 (0.563–1.172)	0.885 (0.627–1.249)	0.916 (0.720–1.164)
High fasting plasma glucose	135 (12.06%)	94 (13.49%)	100 (13.72%)	1099 (13.44%)
	Ref	1.084 (0.832–1.412)	1.022 (0.788–1.325)	1.189 (0.988–1.430)

^1^ Incidence was presented as n (%), and hazard ratios and 95% confidence intervals were calculated using Cox model after adjusting continuous (age, BMI, and energy intake) and categorical (educational level, current drinking status, current smoking status, and physical activity) variables. Non–non: non-consumer both at baseline and follow-up; non–coffee: coffee consumption only at follow-up; coffee–non: coffee consumption only at baseline; coffee–coffee: drinking coffee both at baseline and at follow-up. High triglyceride, serum triglycerides ≥150 mg/dL; Low HDL-cholesterol, serum HDL-cholesterol <40 mg/dL in men and <50 mg/dL in women; Abdominal obesity, waist circumference ≥90 cm in men and ≥80 cm in women; High fasting plasma glucose, fasting plasma glucose ≥100 mg/dL; High blood pressure, systolic blood pressure ≥ 130 mmHg or diastolic blood pressure ≥85 mmHg.

## Data Availability

Restrictions apply to the availability of these data. Data was obtained from National Genome Research Institute and are available at http://koreabiobank.re.kr, accessed on 10 March 2021, with the permission of Nation-al Genome Research Institute.

## Supplementary Materials

The following are available online at https://www.mdpi.com/2072-6643/13/3/920/s1: Table S1: General characteristics of participants based on coffee consumption (cups/day) by sex; Table S2: General characteristics of participants based on the change of coffee consumption pattern by sex; Table S3: Hazard ratios and 95% confidence intervals for MetS according to coffee consumption with or without additives by sex.
