# Supplementary material for: Association of Coffee Consumption and Its Types According to Addition of Sugar and Creamer with Metabolic Syndrome Incidence in a Korean Population from the Health Examinees (HEXA) Study
Source: Nutrients. 2021 Mar 12;13(3):920. doi: 10.3390/nu13030920 (PMC8001379; doi:10.3390/nu13030920)
Supplement: Supplementary file 1 [file nutrients-13-00920-s001.pdf]

nutrients

MDPI

Supplementary Materials

Table S1. General characteristics of participants based on coffee consumption (cups/day) by sex.

	COFFEE CONSUMPTION (CUPS/DAY)				
	0	≤ 1	1–3	> 3	*p* value
Men (N=3,897)					
N (incidence %)	276 (3.99%)	1,071 (4.95%)	1,853 (5.45%)	697 (4.88%)	
Age (years)	55.69 ± 0.50	54.87 ± 0.26	53.75 ± 0.20	50.68 ± 0.29	<.0001
40–49	68 (24.64%)	305 (28.48%)	626 (33.78%)	320 (45.91%)	<.0001
50–59	107 (38.77%)	391 (36.51%)	696 (37.56%)	275 (39.45%)	
60–69	95 (34.42%)	352 (32.87%)	503 (27.15%)	97 (13.92%)	
70–79	6 (2.17%)	23 (2.15%)	28 (1.51%)	5 (0.72 %)	
BMI (kg/m^2)	21.85 ± 0.13	22.53 ± 0.07	22.80 ± 0.05	22.83 ± 0.08	<.0001
< 18.5	23 (8.33%)	44 (4.11%)	52 (2.81%)	16 (2.30%)	<.0001
18.5–25	227 (82.25%)	878 (81.98%)	1,466 (79.11%)	536 (76.90%)	
≥ 25	26 (9.42%)	149 (13.91%)	335 (18.08%)	145 (20.80%)	
Waist circumference (cm)	79.00 ± 0.36	80.04 ± 0.17	80.72 ± 0.13	80.37 ± 0.22	<.0001
Smoking					<.0001
Non-smoker	145 (52.54%)	495 (46.22%)	602 (32.49%)	142 (20.37%)	
Past smoker	110 (39.86%)	410 (38.28%)	752 (40.58%)	225 (32.28%)	
Current smoker	18 (6.52%)	163 (15.22%)	493 (26.61%)	325 (46.63%)	
Physical activity (yes, %)	138 (50.00%)	564 (52.66%)	1,082 (58.39%)	452 (64.85%)	<.0001
Educational level					0.0203
Under middle school	49 (17.75%)	214 (19.98%)	372 (20.08%)	123 (17.65%)	
High school	106 (38.41%)	372 (34.73%)	679 (36.64%)	308 (44.19%)	
College or above	117 (42.39%)	474 (44.26%)	788 (42.53%)	260 (37.30%)	
Drinking status (yes, %)	126 (45.65%)	323 (30.16%)	451 (24.34%)	199 (28.55%)	<.0001
Biomarkers					
TG (mg/dL)	83.42 ± 1.72	86.08 ± 0.95	88.80 ± 0.69	89.38 ± 1.11	0.0632
HDL-C (mg/dL)	54.80 ± 0.69	55.20 ± 0.33	54.76 ± 0.25	53.87 ± 0.39	0.0717
FPG (mg/dL)	87.92 ± 0.42	87.96 ± 0.21	88.58 ± 0.15	87.67 ± 0.27	0.0113
SBP (mmHg)	115.05 ± 0.51	114.62 ± 0.26	114.97 ± 0.20	114.43 ± 0.34	0.5381
DBP (mmHg)	72.53 ± 0.42	72.33 ± 0.21	72.36 ± 0.16	72.02 ± 0.26	0.7748
Women (N=10,725)					
N (incidence %)	1,119 (3.13%)	3,390 (2.92%)	5,234 (3.27%)	9,82 (3.56%)	
Age (years)	52.37 ± 0.21	50.36 ± 0.12	48.53 ± 0.09	46.64 ± 0.18	<.0001
40–49	401 (35.84%)	1600 (47.20%)	3067 (58.60%)	700 (71.28%)	<.0001
50–59	523 (46.74%)	1,427 (42.09%)	1,844 (35.23%)	251 (25.56%)	
60–69	188 (16.80%)	351 (10.35%)	317 (6.06%)	31 (3.16%)	
70–79	7 (0.63%)	12 (0.35%)	6 (0.11%)	0 (0%)	
BMI (kg/m^2)	21.20 ± 0.06	21.72 ± 0.03	21.78 ± 0.03	21.72 ± 0.06	<.0001
< 18.5	97 (8.67%)	179 (5.28%)	224 (4.28%)	38 (3.87%)	<.0001
18.5–25	980 (87.58%)	2,971 (87.64%)	4,557 (87.07%)	846 (86.15%)	
≥ 25	42 (3.75%)	240 (7.08%)	453 (8.65%)	98 (9.98%)	
Waist circumference (cm)	71.59 ± 0.15	72.01 ± 0.08	71.82 ± 0.07	71.51 ± 0.15	0.0283
Smoking					<.0001

Non-smoker	1,107 (98.93%)	3,316 (97.82%)	5,077 (97.00%)	904 (92.06%)	
Past smoker	5 (0.45%)	29 (0.86%)	53 (1.01%)	11 (1.12%)	
Current smoker	5 (0.45%)	30 (0.88%)	78 (1.49%)	65 (6.62%)	
Physical activity (yes, %)	628 (56.12%)	1916 (56.52%)	3,063 (58.52%)	617 (62.83%)	0.0025
Educational level					<.0001
Under middle school	339 (30.29%)	843 (24.87%)	919 (17.56%)	118 (12.02%)	
High school	492 (43.97%)	1,667 (49.17%)	2,638 (50.40%)	456 (46.44%)	
College or above	272 (24.31%)	853 (25.16%)	1,639 (31.31%)	403 (41.04%)	
Drinking status (yes, %)	945 (84.45%)	2,374 (70.03%)	2,979 (56.92%)	523 (53.26%)	<.0001
Biomarkers					
TG (mg/dL)	77.43 ± 0.84	75.59 ± 0.48	74.87 ± 0.38	70.52 ± 0.83	<.0001
HDL-C (mg/dL)	63.41 ± 0.31	63.91 ± 0.18	64.24 ± 0.15	65.20 ± 0.34	0.0734
FPG (mg/dL)	86.13 ± 0.21	86.18 ± 0.12	86.02 ± 0.09	85.25 ± 0.22	0.0059
SBP (mmHg)	111.63 ± 0.30	111.20 ± 0.16	111.16 ± 0.13	110.23 ± 0.30	0.0533
DBP (mmHg)	69.66 ± 0.22	69.58 ± 0.12	69.48 ± 0.10	68.63 ± 0.23	0.0031

[1] *p* value of continuous variables (presented as mean ± standard error) was calculated using general linear models, and the p value of categorical variables (presented as n (%)) was calculated by chi-square test. The same alphabet in same treatment row shows insignificant differences based on Duncan test. Physical inactivity was defined as doing over 30 min exercise less than twice a week. BMI, body mass index;-TG, triglyceride; HDL-C, high-density lipoprotein cholesterol; FPG, fasting plasma glucose; SBP, systolic blood pressure; DBP, diastolic blood pressure.

Table S2. General characteristics of participants based on change of coffee consumption pattern by sex.

	COFFEE CONSUMPTION PATTERN CHANGE				
	Non to Non	Non to Coffee	Coffee to Non	Coffee to coffee	*p* value
Men (N=3,897)					
N (incidence %)	276 (3.99%)	262 (6.87%)	200 (5.50%)	3,159 (5.03%)	
Age (years)	55.69 ± 0.50[a]	54.87 ± 0.53[a]	55.01 ± 0.59[a]	53.28 ± 0.15[b]	0.0006
40–49	68 (24.64%)	79 (30.15%)	59 (29.50%)	1,113 (35.23%)	0.0002
50–59	107 (38.77%)	84 (32.06%)	76 (38.00%)	1,202 (38.05%)	
60–69	95 (34.42%)	93 (35.50%)	61 (30.50%)	798 (25.26%)	
70–79	6 (2.17%)	6 (2.29%)	4 (2.00%)	46 (1.46%)	
BMI (kg/m^2)	21.85 ± 0.13[c]	22.40 ± 0.13[b]	22.44 ± 0.16[b]	22.77 ± 0.04[a]	<.0001
< 18.5	23 (8.33%)	11 (4.20%)	15 (7.50%)	86 (2.72%)	<.0001
18.5–25	227 (82.25%)	220 (83.97%)	163 (81.50%)	2,497 (79.04%)	
≥ 25	26 (9.42%)	31 (11.83%)	22 (11.00%)	576 (18.23%)	
Waist circumference (cm)	79.00 ± 0.36[b]	79.93 ± 0.33[a]	80.57 ± 0.42[a]	80.48 ± 0.10[a]	0.0003
Smoking					
Non-smoker	145 (52.54%)	131 (50.00%)	84 (42.00%)	1,024 (32.42%)	<.0001
Past smoker	110 (39.86%)	99 (37.79%)	89 (44.50%)	1,199 (37.96%)	
Current smoker	18 (6.52%)	31 (11.83%)	26 (13.00%)	924 (29.25%)	
Physical activity (yes, %)	128 (46.38%)	120 (45.80%)	87 (43.50%)	1,217 (38.52%)	0.0120
Educational level					
Under middle school	49 (17.75%)	64 (24.43%)	52 (26.00%)	593 (18.77%)	0.1360
High school	106 (38.41%)	92 (35.11%)	72 (36.00%)	1,195 (37.83%)	
College or above	117 (42.39%)	104 (39.69%)	76 (38.00%)	1,342 (42.48%)	
Drinking status (yes, %)	150 (54.35%)	172 (65.65%)	134 (67.00%)	2,342 (74.14%)	<.0001
Biomarkers					
TG (mg/dL)	83.42 ± 1.72[a]	87.08 ± 1.92[a]	85.26 ± 2.20[a]	88.37 ± 0.53[a]	0.1906
HDL-C (mg/dL)	54.80 ± 0.69[a]	54.44 ± 0.66[a]	54.67 ± 0.70[a]	54.74 ± 0.19[a]	0.8566
FPG (mg/dL)	87.92 ± 0.42[a]	88.38 ± 0.42[a]	88.09 ± 0.49[a]	88.22 ± 0.12[a]	0.8835
SBP (mmHg)	115.05 ± 0.51[a]	114.54 ± 0.56[a]	114.73 ± 0.61[a]	114.79 ± 0.15[a]	0.8464
DBP (mmHg)	72.53 ± 0.42[a]	72.28 ± 0.41[a]	72.08 ± 0.49[a]	72.30 ± 0.12[a]	0.8585
Women (N=10,725)					
N (incidence %)	1,119 (3.13%)	697 (2.73%)	729 (3.16%)	8,180 (3.22%)	
Age (years)	52.37 ± 0.21[a]	51.22 ± 0.26[b]	50.90 ± 0.25[b]	48.62 ± 0.07[c]	<.0001
40–49	401 (35.84%)	290 (41.61%)	316 (43.35%)	4,761 (58.20%)	<.0001
50–59	523 (46.74%)	320 (45.91%)	324 (44.44%)	2,878 (35.18%)	
60–69	188 (16.80%)	86 (12.34%)	85 (11.66%)	528 (6.45%)	
70–79	7 (0.63%)	1 (0.14%)	4 (0.55%)	13 (0.16%)	
BMI (kg/m^2)	21.20 ± 0.06[c]	21.60 ± 0.07[b]	21.65 ± 0.07[ab]	21.78 ± 0.02[a]	<.0001
< 18.5	97 (8.67%)	46 (6.60%)	61 (8.37%)	334 (4.08%)	<.0001
18.5–25	980 (87.58%)	602 (86.37%)	628 (86.15%)	7,144 (87.33%)	

≥ 25	42 (3.75%)	49 (7.03%)	40 (5.49%)	702 (8.58%)	
Waist circumference (cm)	71.59 ± 0.15[b]	71.93 ± 0.18[ab]	72.14 ± 0.17[a]	71.82 ± 0.05[ab]	0.1058
Smoking					
Non-smoker	1,107 (98.93%)	685 (98.28%)	710 (97.39%)	7,902 (96.60%)	0.0019
Past smoker	5 (0.45%)	3 (0.43%)	7 (0.96%)	83 (1.01%)	
Current smoker	5 (0.45%)	5 (0.72%)	10 (1.37%)	158 (1.93%)	
Physical activity (yes, %)	476 (42.54%)	287 (41.18%)	306 (41.98%)	3,208 (39.22%)	0.1098
Educational level					
Under middle school	339 (30.29%)	188 (26.97%)	195 (26.75%)	1,497 (18.30%)	<.0001
High school	492 (43.97%)	344 (49.35%)	371 (50.89%)	4,046 (49.46%)	
College or above	272 (24.31%)	158 (22.67%)	160 (21.95%)	2,577 (31.50%)	
Drinking status (yes, %)	174 (15.55%)	178 (25.54%)	186 (25.51%)	3,366 (41.15%)	<.0001
Biomarkers					
TG (mg/dL)	77.43 ± 0.84[a]	77.13 ± 1.08[a]	76.61 ± 1.04[ab]	74.30 ± 0.31[b]	0.0129
HDL-C (mg/dL)	63.41 ± 0.31[a]	63.69 ± 0.39[a]	63.62 ± 0.38[a]	64.32 ± 0.12[a]	0.5571
FPG (mg/dL)	86.13 ± 0.21[a]	85.85 ± 0.28[a]	85.86 ± 0.28[a]	86.02 ± 0.08[a]	0.7536
SBP (mmHg)	111.63 ± 0.30[a]	111.45 ± 0.37[a]	111.38 ± 0.35[a]	111.02 ± 0.11[a]	0.4572
DBP (mmHg)	69.66 ± 0.22[a]	69.81 ± 0.27[a]	69.33 ± 0.27[a]	69.40 ± 0.08[a]	0.3854

[1] *p* value of continuous variables (presented as mean ± standard error) was calculated using general linear models, and the *p* value of categorical variables (presented as n (%)) was calculated by chi-square test. The same alphabet in same treatment row shows insignificant differences based on Duncan test. Non-non: non-consumer both at baseline and follow-up; non-coffee: drinking coffee only at follow-up; coffee-non: drinking coffee only at baseline; coffee-coffee: drinking coffee both at baseline and at follow-up. Physical inactivity was defined as doing over 30 min exercise less than twice a week. BMI, body mass index; TG, triglyceride; HDL-C, high-density lipoprotein cholesterol; FPG, fasting plasma glucose; SBP, systolic blood pressure; DBP, diastolic blood pressure.

Table S3. Hazard ratios and 95% confidence intervals for MetS according to coffee consumption with or without additives by sex[1].

	Coffee type				
	No	Black	3-in-1	Others	
				additives ≤ 1 teaspoon	additives > 1 teaspoon
Men (N=3,897)	276 (7.08%)	90 (2.31%)	610 (15.65%)	1,093 (28.05%)	1,828 (46.91%)
MetS	Ref	0.679 (0.180–2.563)	0.841 (0.398–1.778)	1.015 (0.524–1.964)	0.692 (0.342–1.398)
Abdominal obesity	Ref	0.941 (0.357–2.480)	0.955 (0.521–1.751)	0.908 (0.522–1.578)	1.107 (0.632–1.937)
High triglyceride	Ref	1.479 (0.744–2.939)	0.968 (0.594–1.578)	1.096 (0.705–1.704)	0.904 (0.570–1.432)
High blood pressure	Ref	1.442 (0.916–2.270)	0.812 (0.591–1.115)	0.816 (0.619–1.076)	0.776 (0.579–1.040)
Low HDL-cholesterol	Ref	1.111 (0.433–2.849)	0.358 (0.174–0.736)	0.449 (0.253–0.796)	0.409 (0.222–0.752)
High fasting plasma glucose	Ref	0.966 (0.600–1.556)	0.636 (0.470–0.860)	0.687 (0.530–0.891)	0.749 (0.571–0.984)
Women (N=10,725)	1,119 (10.43%)	548 (5.11%)	1,130 (10.54%)	4,401 (41.03%)	3,527 (32.89%)
MetS	Ref	0.556 (0.281–1.099)	0.549 (0.319–0.945)	0.671 (0.450–1.000)	0.825 (0.536–1.269)
Abdominal obesity	Ref	0.874 (0.662–1.155)	0.992 (0.796–1.236)	0.941 (0.788–1.124)	1.034 (0.854–1.254)
High triglyceride	Ref	1.027 (0.680–1.551)	0.855 (0.618–1.184)	0.809 (0.633–1.033)	1.000 (0.761–1.314)
High blood pressure	Ref	1.172 (0.879–1.564)	0.918 (0.724–1.163)	0.824 (0.684–0.993)	0.907 (0.740–1.112)
Low HDL-cholesterol	Ref	1.157 (0.750–1.786)	0.950 (0.673–1.340)	0.787 (0.605–1.025)	1.157 (0.867–1.545)
High fasting plasma glucose	Ref	1.367 (1.011–1.849)	0.979 (0.759–1.263)	1.000 (0.820–1.219)	1.077 (0.866–1.338)

[1] Hazard ratios and 95 % confidence intervals were calculated using Cox model after adjusting for continuous (age, BMI, and energy intake) and categorical (educational level, current drinking status, current smoking status, and physical activity) variables. MetS, metabolic syndrome; BMI, body mass index; HDL, high-density lipoprotein. High triglyceride, serum triglycerides ≥ 150 mg/dL; Low HDL-cholesterol, serum HDL-cholesterol < 40 mg/dL in men and < 50 mg/dL in women; Abdominal obesity, waist circumference ≥ 90 cm in men and ≥ 80 cm in women; High fasting plasma glucose, fasting plasma glucose ≥ 100 mg/dL; High blood pressure, systolic blood pressure ≥ 130 mmHg or diastolic blood pressure ≥ 85 mmHg. additives ≤ 1 teaspoon: adding sugar or creamer no more than 1 teaspoon. additives > 1 teaspoon: adding sugar or creamer over than 1 teaspoon.
